# Does a fluoro-assisted direct anterior approach for total hip arthroplasty pose an excessive risk of radiation exposure to the surgeon?

**DOI:** 10.1051/sicotj/2020004

**Published:** 2020-02-18

**Authors:** Yuta Jinnai, Tomonori Baba, Xu Zhuang, Hiroki Tanabe, Sammy Banno, Taiji Watari, Yasuhiro Homma, Kazuo Kaneko

**Affiliations:** 1 Department of Orthopaedic Surgery, Juntendo University School of Medicine 113-0033 Tokyo Japan

**Keywords:** Total hip arthroplasty, direct anterior approach, radiation exposure, fluoroscopy

## Abstract

*Introduction*: Intraoperative fluoroscopy can be easily used because patients are placed in the supine position during total hip arthroplasty via direct anterior approach (DAA-THA) to reduce complications. However, the cumulative level of radiation exposure by intraoperative fluoroscopy increases as the annual number of cases increases, increasing the risk of influencing the health of both the patients and medical workers. The objective of the study was to compare the radiation exposure time of DAA-THA with osteosynthesis and to determine if the level of radiation exposure exceeded safety limits. *Material and methods*: DAA-THA was performed in 313 patients between January 2016 and July 2018 and 60 patients with proximal femoral fracture were treated with osteosynthesis. The intraoperative fluoroscopy time was retrospectively surveyed and compared between these two groups. A total of eight surgeons operated DAA-THA employing the same procedure using a traction table. A total of nine surgeons operated osteosynthesis and fluoroscopy was appropriately used during reduction and implant insertion. *Results*: The mean operative time of DAA-THA was 103.3 min and that of osteosynthesis was 83.3 min, showing a significant difference (*p* < 0.05). The mean intraoperative fluoroscopy time was 0.83 min (SD ± 0.68) in DAA-THA and 8.91 min (SD ± 8.34) in osteosynthesis showing a significant difference (*p* < 0.05). *Conclusions*: The intraoperative exposure level was significantly lower and the fluoroscopy time was significantly shorter in DAA-THA than in osteosynthesis for proximal femoral fracture. It was clarified that the annual cumulative radiation exposure level in DAA-THA does not exceed the tissue dose limit.

## Introduction

Total hip arthroplasty (THA) is an effective surgical method for the reduction of hip joint pain, functional recovery, and improvement of quality of life (QOL) [1, 2]. There are various approaches for THA and one of these, the direct anterior approach (DAA), has recently been attracting attention as an intermuscular and inter-innervated area approach [3]. The characteristic of DAA is low-invasiveness and soft tissue can be conserved, so that postoperative recovery is fast and it is associated with a low dislocation rate and reduction of postoperative pain, for which a favorable postoperative outcome can be expected [4, 5]. However, it has been occasionally reported that the risk of complications is high early after the introduction of DAA for THA, such as failure of component placement and femoral fracture, and the presence of a learning curve has been pointed out [6]. One of the measures to reduce these complications is intraoperative fluoroscopy. In DAA, intraoperative fluoroscopy can be easily used because patients are placed in the supine position during surgery [7]. Appropriate use of intraoperative fluoroscopy may improve not only the complication-reducing effect of total hip arthroplasty via direct anterior approach (DAA-THA) but also the postoperative outcome through accurate evaluation of leg length discrepancy and offset [8–10].

The acceptable radiation exposure level of orthopedists is controversial. The use of intraoperative fluoroscopy is essential for orthopedists to perform osteosynthesis for proximal femoral fracture. Generally, the exposure level during osteosynthesis for proximal femoral fracture applied in the same region as THA is higher than that during osteosynthesis in other regions, so that reduction of the exposure level is necessary [11].

Thus, we hypothesized that the intraoperative exposure level during DAA-THA is far lower than that during osteosynthesis for proximal femoral fracture. The objective of the study was to compare the radiation exposure time of DAA-THA with osteosynthesis and to determine if the level of radiation exposure exceeded safety limits.

## Material and methods

### Patients

DAA-THA was performed in 313 patients between January 2016 and July 2018. During the same period, 60 patients with proximal femoral fracture were treated with osteosynthesis (neck fractures: 14, trochanteric fractures: 38, subtrochanteric fractures: 8). The intraoperative fluoroscopy time was retrospectively surveyed and compared between these two groups. The study was approved by the Central Ethical Review Board of our institution (entry number 18-219 approved on December 28, 2018). Patient information including the age, gender, height, weight, body mass index (BMI), and diagnosis were recorded.

### Surgery

A total of eight surgeons operated DAA-THA employing the same procedure using a traction table. A surgeon (T.B.) familiar with DAA supervised surgery in all cases. Intraoperative fluoroscopy was performed in all cases of DAA-THA. The timing of intraoperative fluoroscopy was: (1) final reaming of the acetabular roof, (2) placement of an acetabular cup, (3) insertion of a trial stem, (4) confirmation of leg length discrepancy after temporary reduction, and (5) after the final implant placement. The implant used was cementless in all cases and several screws were added to the acetabular component cup on the judgment of the operator.

A total of nine surgeons operated osteosynthesis and a surgeon (T.W.) familiar with osteosynthesis supervised surgery in all cases. Intraoperative fluoroscopy was used in all cases. A traction table was used in all cases and fluoroscopy was appropriately used during reduction and implant insertion. For the fluoroscopic apparatus, surgical X-ray apparatus OEC 9900 Elite (GE Healthcare Japan, Tokyo, Japan) was used. The surgeons performed surgery wearing a lead apron as a protective clothing.

### Measurement of radiation exposure

**Figure 1:**
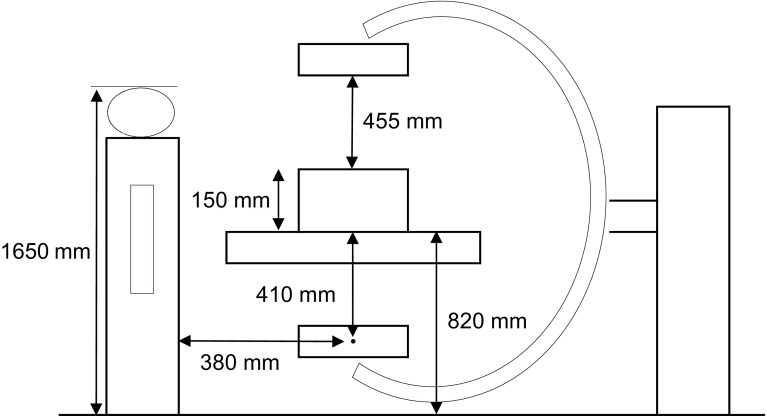
Simulation of DAA-THA by a phantom.

For the exposure level of the operators, the data measured by the fluoroscopic apparatus were summed using the area dosimeter display values immediately after surgery. The fluoroscopy time was retrospectively investigated in the irradiation record of the fluoroscopic apparatus. Since the fluoroscopy time was surveyed in this study and the actual radiation dose was unclear, as shown in Figure 1, phantoms were arranged the same as those in actual surgery and the radiation dose was measured in the thyroid region outside the protector, chest inside the protector, and eye region of the operator using a pocket dosimeter, PDM-127B-SZ (Hitachi health care, Tokyo, Japan), and dosimeter for the lens, DOSIRIS™ (IRSN, France) [12].

### Statistical analysis

Statistical analysis was performed using IBM SPSS Statistics 25 for Macintosh (SPSS Inc., Chicago, IL, USA). Independent-samples Student’s *t*-test was used for continuous variables, and the chi-squared test was used for dichotomous variables. *p* < 0.05 was regarded as significant in all analyses.

## Results

### Patient information

Table 1 shows the patient information. The age, gender, weight, and BMI were significantly different between the two groups, but no significant difference was noted in the height.

**Table 1:** Patient characteristics.

Measurements	DAA-THA	Osteosynthesis for proximal femoral fractures	*p* value
Patients (number)	313	60	
Age (years)	67.8 (27–92)	76.2 (53–100)	<0.05*
Gender (male/female)	69(22.0%)/244(78.0%)	25(41.7%)/35(58.3%)	<0.05*
Height (cm)	155.4 (128.5–187.4)	154.4 (120.0–182.0)	0.521
Weight (kg)	56.7 (29.0–113.5)	50.2 (34.0–85.0)	<0.05*
BMI (kg/m^2^)	23.4 (12.1–41.4)	21.1 (14.4–37.4)	<0.05*
Pre-operative diagnosis			
Osteoarthritis	231 (73.8%)		
Rheumatoid arthritis	2 (0.6%)		
Avascular necrosis of the femoral head	29 (9.3%)		
Femoral neck fracture	51 (16.3%)	14 (23.3%)	
Trochanteric fracture		38 (63.3%)	
Subtrochanteric fracture		8 (13.3%)	

DAA-THA: total hip arthroplasty via direct anterior approach.

*Significant difference.

### Surgical information

Table 2 shows the surgical information. The mean operative time of DAA-THA was 103.3 min and that of osteosynthesis was 83.3 min, showing a significant difference (*p* < 0.05). The intraoperative blood losses were 472.9 mL and 86.0 mL, respectively, being significantly smaller in osteosynthesis. The mean intraoperative fluoroscopy time was 0.83 min (SD ± 0.68) in DAA-THA and 8.91 min (SD ± 8.34) in osteosynthesis (neck fractures: 6.14 min, trochanteric fractures: 7.81 min, subtrochanteric fractures: 18.98 min), showing a significant difference (*p* < 0.05). The mean exposure level per one DAA-THA determined using the results of the investigation using phantoms and mean operative time of the surgeries was 11.09 μSv in the thyroid region outside the protector, 0.21 μSv on the chest inside the protector, and 5.55 μSv in the eye region. In osteosynthesis, the mean exposure level was 118.58 μSv in the thyroid region outside the protector, 2.29 μSv on the chest inside the protector, and 59.38 μSv in the eye region.

**Table 2:** Operation information.

Measurements	DAA-THA	Osteosynthesis for proximal femoral fractures	*p* value
Operative side (right/left)	167(53.4%)/146(46.6%)	22(36.7%)/38(63.3%)	<0.05*
Operation time (min)	103.3 (52–274)	83.3 (28–206)	<0.05*
Blood loss volume (mL)	472.9 (50–2498)	86.0 (3–500)	<0.05*
Radiation time (min)	0.83 (0.1–3.0)	8.91 (0.5–39.8)	<0.05*
Femoral neck fracture		6.14 (2.8–18.0)	
Trochanteric fracture		7.81 (0.5–20.3)	
Subtrochanteric fracture		18.98 (3.3–39.8)	
Radiation dose (μSv)			
Thyroid (outside of a lead apron)	11.09 (1.33–39.94)	118.58 (6.66–529.87)	
Sternal notch (inside of a lead apron)	0.21 (0.03–0.77)	2.29 (0.13–10.22)	
Eye	5.55 (0.67–20.00)	59.38 (3.33–265.33)	

DAA-THA: total hip arthroplasty via direct anterior approach.

*Significant difference.

## Discussion

Accurate implant placement in THA is one of the most important factors to improve the postoperative outcome. It has recently been reported that computed tomography (CT) navigation and simple navigation are useful for accurate implant placement, but these are not generally used due to the cost [7]. In contrast, fluoroscopic apparatus is installed in most facilities capable of performing orthopedic surgery and supported as a useful tool for DAA-THA. Radiograms have been used at medical practice sites since it was discovered by Röntgen in 1895. After the intraoperative fluoroscopy method spread early in the 1980s, opportunities for its use has been increasing yearly, but the cumulative level of radiation exposure by intraoperative fluoroscopy increases as the annual number of cases increases, increasing the risk of influencing the health of both the patients and medical workers [13]. Operation is performed staying close to the radiation source in many cases of fluoroscopic surgery, and radiation exposure is unavoidable for many orthopedists. The objective of this study was to quantify the intraoperative exposure level of surgeons and intraoperative fluoroscopy time and compared these between DAA-THA and osteosynthesis for proximal femoral fracture.

Studies on radiation exposure in DAA-THA have been occasionally reported, but the detection method of the radiation exposure level varied and was not uniform (Table 3) [14–17]. Thus, we compared only the intraoperative fluoroscopy time. The intraoperative fluoroscopy time in DAA-THA was 0.23–0.59 min in preceding studies, being shorter than that (0.83 min) in our study. This may have been due to compliance with the protocol of the use of intraoperative fluoroscopy standardized in this study, which prolonged the intraoperative fluoroscopy time. The mean intraoperative fluoroscopy time in osteosynthesis for proximal femoral fracture was 0.53–11.8 min [11, 18, 19], being equivalent to that (8.91 min) in our study. Radiation exposure in DAA-THA was far lower than that in routinely performed osteosynthesis for proximal femoral fracture, demonstrating that the risk of unhealthy radiation exposure to the surgeon during DAA-THA is very low. The intraoperative fluoroscopy time was investigated and compared involving a greater number of DAA-THA cases (313) than those (50–157) in the preceding studies, and the total number of operators was 8, also being greater. In addition, all patients received surgery in the same facility. This indicates that the averaged intraoperative fluoroscopy time was measured along with actual clinical practice, unlike data of surgeries performed by a specified surgeon or performed at multiple facilities. These are differences from preceding studies and novelty can be claimed. Furthermore, to our knowledge, there is no study comparing intraoperative fluoroscopy time of osteosynthesis for proximal femoral fracture and DAA-THA, therefore that point can also claim the novelty of this study.

**Table 3:** The comparison with previous studies on exposure time.

Measurements			Radiation time		Radiation dose	
Present study	Surgeon	Thyroid (outside of a lead apron)	0.83 (0.1–3.0)	min	11.09 (1.33–39.94)	μSv
		Sternal notch (inside of a lead apron)			0.21 (0.03–0.77)	μSv
		Eye			5.55 (0.67–20.00)	μSv
Curtin et al. [14]	Patient	The individual C-arm fluoroscopy units	23.74 (11.3–61.7)	sec	2.97 ± 1.63 (0.29–9.83)	mGy
Pomeroy et al. [15]	Surgeon	A helmet-mounted dosimeter	15.06 ± 6.73	sec	2.00 ± 1.3	mGy
					0.217	mrem
	Patient	The dosimeter badge placed near the eye			0.0022	mGy
McArthur et al. [16]	Surgeon	A dosimeter over the lead in the chest area	0.59	min	10	mrem
McNabb et al. [17]	Surgeon	Fluoroscopic machine	13.72 (6.7–28.7)	sec	178 (54–526)	mrem

In the radiation exposure index established by the International Commission on Radiological Protection (ICRP), the annual dose limit for individuals is set for each organ [20]. The mean annual dose limit of 5 years is specified at 20 mSv (100 mSv in 5 years) with an effective dose per year not exceeding 50 mSv. In addition, the tissue dose limit considered able to prevent various tissue damages is specified at 500 mSv for the skin (500,000 μSv) and 150 mSv per year (150,000 μSv) for the eye lens. Since the mean exposure level per one application of DAA-THA was 11.09 μSv in the thyroid region outside the protector and 5.55 μSv in the eye region, to exceed the tissue dose limit, more than about 45,000 and 27,000 surgeries per year are necessary for the skin and eye, respectively. The mean exposure level per one application of DAA-THA was 0.21 μSv on the chest inside the protector, so that more than about 238,000 surgeries are necessary to reach the limit when the operator wears a protector. Thus, DAA-THA is a very safe surgery with regard to radiation exposure. However, the hands operating the surgical field are not protected, being exposed compared with exposure of the trunk wearing protective clothing. Although the exposure level does not exceed the tissue dose limit of the hands and legs, 500 mSv (500,000 μSv), sufficient attention should be paid to exposure of the hands and fingers.

Radiation disorders include acute damage occurring upon exposure to a high dose and late disorder developing several years or decades after exposure to a low dose of radiation. In late disorder, a small dose exposure may induce cancer and cataract [21, 22]. Development of cataract is of big concern for some surgeons, especially those who use intraoperative fluoroscopy in routine practice (orthopedic trauma surgeons, vascular surgeons, and interventional cardiologists). According to ICRP, the threshold radiation exposure to induce cataract is 0.5 Gy (400,000 μSv) [23]. In our study, the intraoperative exposure level in the eye region was 5.55 μSv, clarifying that the threshold for cataract is reached after performing more than about 72,000 surgeries of DAA-THA even though the operator does not wear an eye protector. So, practically, the risk for cataract is as low as possible. In our additional survey, the exposure level inside the protector was much lower than that outside the protector in each group. It has also been reported that exposure to scattered radiation can be reduced by 90% or more by wearing a lead apron and thyroid shield [13, 24], suggesting that the shielding effect of the protectors is reliable. It is important to make efforts to reduce the exposure level of operators by wearing a lead apron and thyroid shield in surgery using fluoroscopy. Furthermore, Muller et al. stated that fluoroscopy should not be continuously used during surgery and it should be used only when it is necessary, termed “pulse fluoroscopy” [25]. In DAA-THA, continuous fluoroscopy is unnecessary because fluoroscopy is used to confirm the position of an acetabular cup placement and evaluate leg length discrepancy and offset, and intermittent short-time fluoroscopy may be sufficient.

Regarding limitations, only the fluoroscopy time recorded by the fluoroscopic apparatus was compared because the operators did not use a personal radiation dosimeter. However, sufficient data may have been acquired because the fluoroscopy time recorded by the fluoroscopic apparatus and radiation dose determined by simulation using phantoms were analyzed.

## Conclusions

The intraoperative exposure level was significantly lower and the fluoroscopy time was significantly shorter in DAA-THA than in osteosynthesis for proximal femoral fracture. It was clarified that the annual cumulative radiation exposure level in DAA-THA does not exceed the tissue dose limit.

## Conflict of interest

The authors declare that they have no conflict of interest.

## Ethical approval

Study is approved by our institutional review board.
